# TRPV4 regulates calcium homeostasis, cytoskeletal remodeling, conventional outflow and intraocular pressure in the mammalian eye

**DOI:** 10.1038/srep30583

**Published:** 2016-08-11

**Authors:** Daniel A. Ryskamp, Amber M. Frye, Tam T. T. Phuong, Oleg Yarishkin, Andrew O. Jo, Yong Xu, Monika Lakk, Anthony Iuso, Sarah N. Redmon, Balamurali Ambati, Gregory Hageman, Glenn D. Prestwich, Karen Y. Torrejon, David Križaj

**Affiliations:** 1 Department of Ophthalmology & Visual Sciences, Moran Eye Institute, University of Utah School of Medicine, Salt Lake City, UT 84132, USA; 2 Interdepartmental Program in Neuroscience, University of Utah School of Medicine, Salt Lake City, UT 84132, USA; 3 Department of Medicinal Chemistry, University of Utah School of Medicine, Salt Lake City, UT 84132, USA; 4 Center for Translational Medicine, University of Utah School of Medicine, Salt Lake City, UT 84132, USA; 5 Glauconix, Inc., Albany, NY, USA; 6 Department of Neurobiology & Anatomy, University of Utah School of Medicine, Salt Lake City, UT 84132, USA; 7 Department of Bioengineering, University of Utah, Salt Lake City, UT 84112, USA

## Abstract

An intractable challenge in glaucoma treatment has been to identify druggable targets within the conventional aqueous humor outflow pathway, which is thought to be regulated/dysregulated by elusive mechanosensitive protein(s). Here, biochemical and functional analyses localized the putative mechanosensitive cation channel TRPV4 to the plasma membrane of primary and immortalized human TM (hTM) cells, and to human and mouse TM tissue. Selective TRPV4 agonists and substrate stretch evoked TRPV4-dependent cation/Ca^2+^ influx, thickening of F-actin stress fibers and reinforcement of focal adhesion contacts. TRPV4 inhibition enhanced the outflow facility and lowered perfusate pressure in biomimetic TM scaffolds populated with primary hTM cells. Systemic delivery, intraocular injection or topical application of putative TRPV4 antagonist prodrug analogs lowered IOP in glaucomatous mouse eyes and protected retinal neurons from IOP-induced death. Together, these findings indicate that TRPV4 channels function as a critical component of mechanosensitive, Ca^2+^-signaling machinery within the TM, and that TRPV4-dependent cytoskeletal remodeling regulates TM stiffness and outflow. Thus, TRPV4 is a potential IOP sensor within the conventional outflow pathway and a novel target for treating ocular hypertension.

Intraocular pressure (IOP) is the most significant and only treatable risk factor for glaucomas, with the risk of progression decreasing ~10% for every mm Hg of IOP reduction^1^. Anti-glaucoma medications aim to lower IOP-induced retinal damage by decreasing the production of aqueous fluid in the anterior eye (‘inflow’) or increasing its drainage through the secondary, ‘uveoscleral’ outflow pathway^2^,^3^. However, the main route of aqueous fluid drainage and the predominant site of the abnormal resistance to fluid outflow in glaucoma is the ‘conventional’ pathway formed by the juxtacanalicular trabecular meshwork (TM) which channels aqueous flow into the Schlemm’s canal^4^,^5^,^6^,^7^,^8^. The absence of topical treatments targeting the conventional outflow pathway currently represents a major impediment for treating glaucoma^2^.

The hydraulic conductivity of conventional fluid outflow is affected by the mechanical microenvironment that controls the shape, volume, contractility and second messenger signaling of TM cells. Abnormal strain may pathologically increase tissue resistance to aqueous drainage, thereby elevating IOP and potentially facilitating optic nerve damage and blindness in susceptible individuals^4^,^5^. TM cells respond to mechanical stretching caused by increased IOP with altered gene expression, intracellular signaling and increased turnover of extracellular matrix (ECM) proteins^6^,^7^,^8^. If IOP elevations are sustained, they induce compensatory increases in the rigidity of ECM and the cells’ cytoskeleton, which may further obstruct fluid outflow^3^,^9^,^10^. Although it is obvious that mechanosensory mechanisms employed by TM regulate IOP homeostasis and that their chronic overactivation drives progressive remodeling of the biomechanical environment in disease, the identity and function of these mechanosensor(s) remain largely unknown.

There are several indications that elevated IOP mechanically strains TM cells (e.g, via stretching ECM), which perturbs Ca^2+^ homeostasis and restructures the architecture of the ECM/cytoskeleton: (a) mechanical stress increases [Ca^2+^]_TM_^11^ and triggers the formation of actin stress fibers^12^; (b) [Ca^2+^]_TM_ is elevated in eyes with primary open angle glaucoma (POAG) compared to control eyes^13^; and (c) agents that elevate [Ca^2+^]_TM_ (endothelin-1, bradykinin) increase the TM resistance to aqueous outflow^9^, whereas actin depolymerizers increase the conventional ‘outflow facility’ and lower IOP^10^,^14^. Despite the central role for mechanotransduction in TM Ca^2+^ signaling and cytoskeletal remodeling, the molecular mechanisms that mediate force coupling and their role in IOP regulation remain poorly understood. Here, we identify a key force sensor as TRPV4 (transient receptor potential vanilloid 4), a nonselective cation channel that regulates osmo-, thermo-, mechanosensation and nociception across the animal kingdom^15^,^16^,^17^,^18^,^19^,^20^,^21^. Our results show that TM TRPV4 represents a crucial link between membrane stretch, Ca^2+^ signals and cytoskeletal reorganization, and that TRPV4 activation is required for persistent IOP elevation in an animal model of glaucoma. We developed putative TRPV4 antagonist prodrug analogs that are effective in lowering IOP and protecting downstream retinal neurons. Moreover, we found that TRPV4 inhibition directly increases the outflow facility in biomimetic scaffolds populated with primary human TM cells. Hence, this work reveals a novel role for TRPV4-mediated Ca^2+^ influx in TM function and points toward a new viable alternative for the therapeutic control of the conventional outflow pathway.

## Results

### TRPV4 is expressed in cultured TM and *in vivo* TM

**Figure 1:**
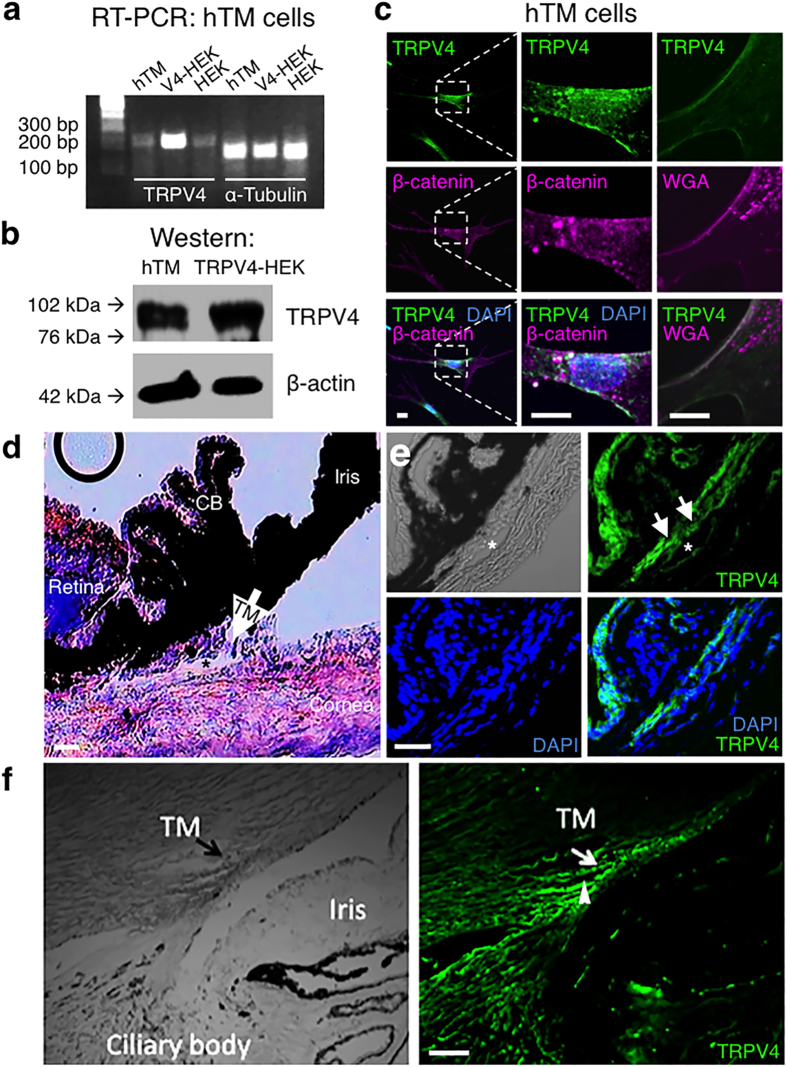
TRPV4 is expressed in human TM cells and localizes to the plasma membrane. (**a**) *Trpv4* mRNA (217 bp amplicon) and α-tubulin from hTM, TRPV4-overexpressing HEK293 cells and control HEK293 cells. (**b**) TRPV4 protein (~95 kDa) is expressed in cultured hTM cells and TRPV4-overexpressing HEK293 cells. (**c**) In hTM cells TRPV4 (green) colocalizes with the *adherens* junction marker β-catenin (magenta) or the plasma membrane marker WGA (magenta). DAPI labels cell nuclei (blue). Scale bars = 10 μm. (**d**) Mouse eye section stained with H & E. TM denoted by arrow. Schlemm’s canal at asterisk. CB = ciliary body. Scale bar = 20 μm. (**e**) Mouse eye section immunostained for TRPV4. TM denoted by arrows. Schlemm’s canal at asterisk. Scale bar = 50 μm. Experiments were performed ≥3 times. (**f**) Anterior chamber in the human eye (left), labeled with the TRPV4 antibody (right). TRPV4 expression includes TM (arrow) and putative endothelial cells within the Schlemm’s canal (arrowhead). Scale bar = 100 μm.

**Figure 2:**
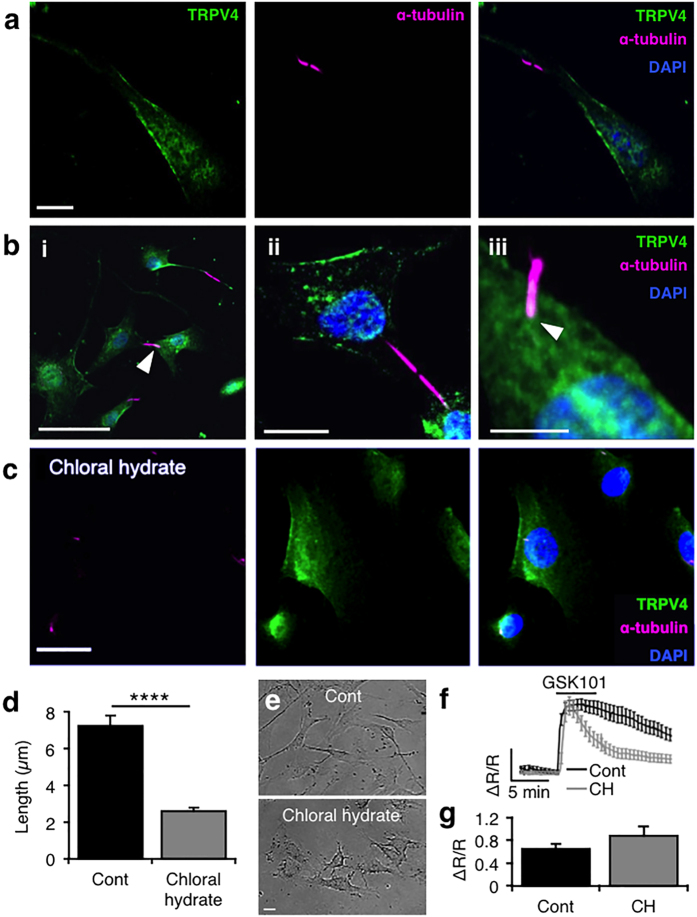
TRPV4 signals are largely independent of primary cilia in hTM cells. (**a**) Representative image from an hTM cell labeled with anti-TRPV4 (AlexaFluor 488 nm) and anti-α-tubulin antibodies (Alexa Fluor 594 nm) shows TRPV4-ir within patches of the plasma membrane, whereas the cilium is immunonegative for TRPV4. Scale bar = 10 μm. (**b**) Images from hTM (**i**) and pTM (**ii, iii**) cells show that TRPV4 may be localized to the ciliary base in a subset of TM cells (arrowheads). Scale bars = 50 μm (**i**), 10 μm (**ii**) and 5 μm (**iii**). (**c,d**) Chloral hydrate (CH; 4 mM; 24 hrs) reduced the number and length of primary cilia within hTM cells and uncoupled ciliary bridges between adjacent cells. (**e**) Brightfield images of hTM cell morphology indicate that chloral hydrate is not TM-toxic. Scale bars = 20 μm. (**f**,**g**) CH post-peak [Ca^2+^]_i_ plateau phase but has no effect on the amplitude of GSK101-evoked [Ca^2+^]_i_ signals.

The TM origin of cultured human TM (hTM) cells was confirmed by the expression of TM-specific genes and upregulation of *Myoc* (myocilin) in response to dexamethasone (DEX) treatment (Supplementary Fig. S1). We analyzed TRPV4 expression, localization and function in cultured hTM and primary human TM (pTM) cells and in mouse and human eyes *in vivo*. RT-PCR and Western blot analysis showed *Trpv4* mRNA and protein expression in hTM cells (Fig. 1a,b). A TRPV4 antibody, validated in human and *Trpv4*^−/−^ mouse tissues^22^,^23^,^24^, labeled the plasma membrane of every examined hTM and pTM cell (Figs 1c and 2a–c). Double labeling for the plasma membrane marker WGA (wheat germ agglutinin) or the *adherens* junction marker β-catenin showed TRPV4-immunoreactivity (TRPV4-ir) to be predominantly expressed across patches of the plasma membrane (Fig. 1c), whereas co-staining with the primary ciliary marker acetylated α-tubulin only revealed the presence of ciliary TRPV4-ir in a subset of hTM cells (24/95 cells; 25.3%) and pTM cells (6/28 cells; 21.4%) (Fig. 2a,b). Hematoxylin and eosin staining of a mouse eye section is shown to illustrate the anatomy of relevant ocular structures (Fig. 1d). The TRPV4 antibody also labeled TM in mouse (Fig. 1e) and human (Fig. 1f) eyes. Additionally, presumed endothelial cells lining the Schlemm’s canal (asterisk in Fig. 1d,e; arrowhead in Fig. 1f) and nonpigmented epithelial cells of the ciliary body exhibited TRPV4-ir (Fig. 1e,f).

### TM TRPV4 channels are functional, mediating sustained inward currents and [Ca^2+^]_i_ elevations

**Figure 3:**
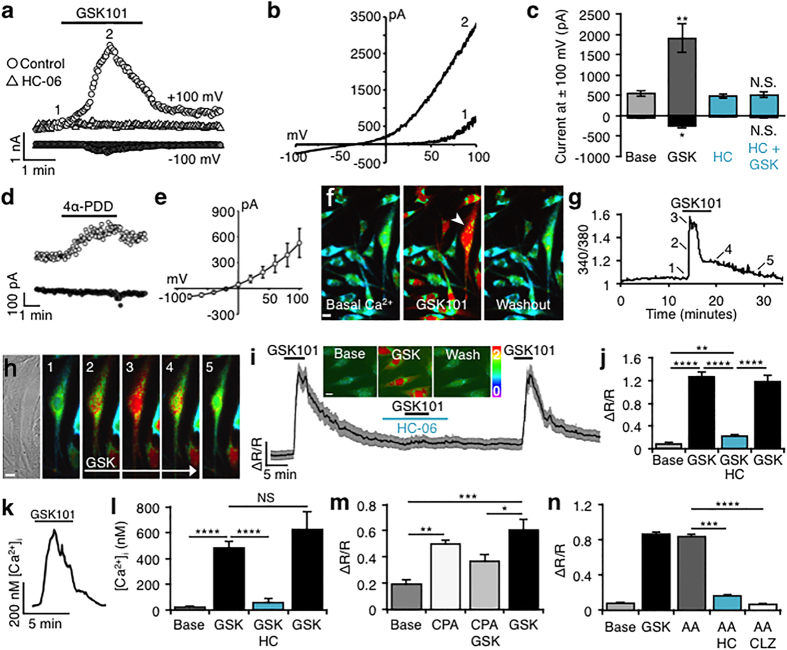
TRPV4 is functional, mediating transmembrane current and [Ca^2+^]_i_ elevations in hTM cells. (**a**) Time course of GSK101-evoked currents at +100 mV (*open circles*) and −100 mV (*closed circles*). Block by HC-06 (1 μM) shown as *open and closed triangles*, respectively. Acquisitions of I-V data denoted by numerals “1” and “2”. (**b**) Representative I-V curves of pre-agonist (1) and GSK101-induced (2) responses from (**a**). (**c**) Cumulative data for GSK101 (n = 9) and GSK101+ HC-06 (n = 12) currents at ±100 mV. (**d**) Time-course for 4α-PDD (1 μM) -evoked currents at V_h_ = − 100 mV *(filled symbols)* and +100 mV *(open symbols).* (**e**) Averaged I-V relationship of 4α-PDD -induced currents obtained by subtraction of the pre- application response from peak response (n = 9). (**f**) Wide-field view of Fura-2-loaded hTM cells stimulated with GSK101 and washout. Scale bar = 10 μm. (**g**) Time course of the [Ca^2+^]_i_ signal for the arrowhead-marked cell in (**f**). (**h**) Transmitted & time lapse images from the cell marked in (**f**). Scale bar = 10 μm. (**i,j**) hTM. GSK101 (25 nM) elevates [Ca^2+^]_i_, inhibition by HC-06 and washout. Gray bars denote SEM. *Inset*: representative ratiometric images. Scale bar = 10 μm. (**k**) Time course of calibrated [Ca^2+^]_i_ increase in a pTM cell. (**l**) Quantification of [Ca^2+^]_i_ responses from pTM cells in the presence/absence of HC-06 washout. (**m**) hTM. CPA (5 μM) elevates [Ca^2+^]_i_ levels above baseline fluctuations, resulting in suppression (P < 0.05) of the TRPV4 response. (**n**) hTM. AA (50 μM) evokes responses that are blocked by HC-06 (1 μM) and CTZ (10 μM).

**Figure 4:**
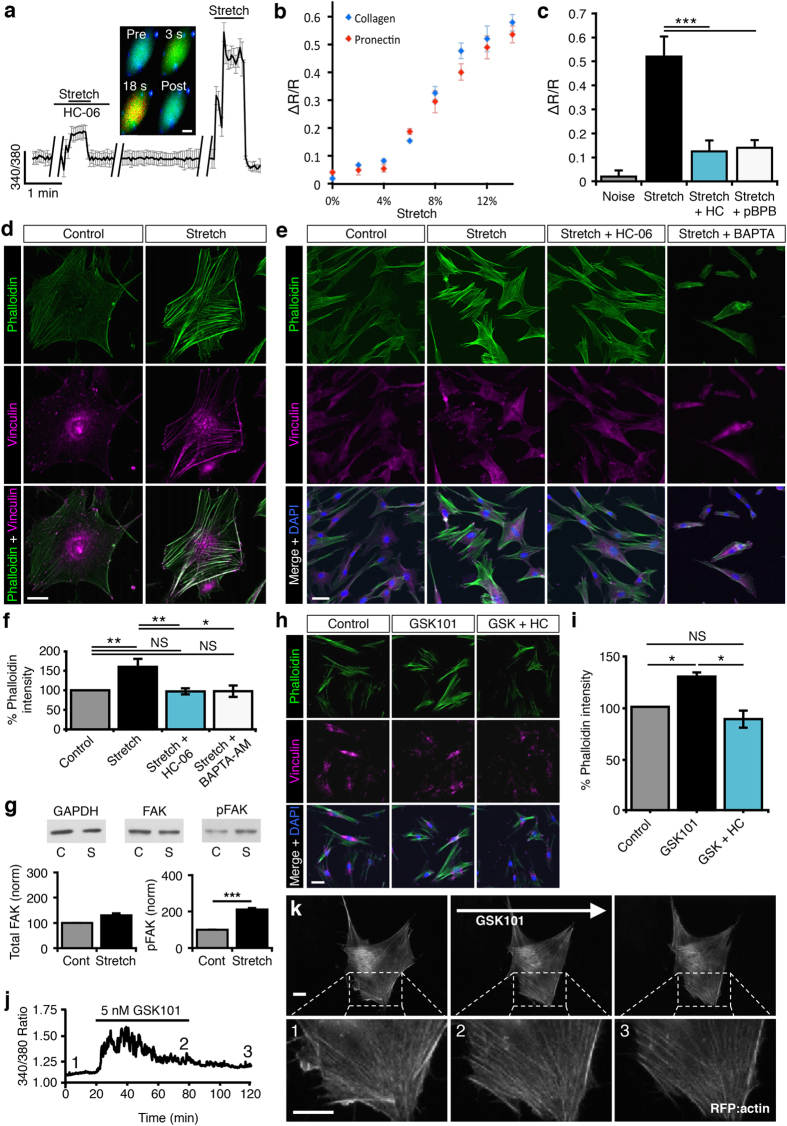
TRPV4 and stretch-dependent Ca^2+^ influx induce cytoskeletal reorganization. (**a**) Stretch (14%) elevates [Ca^2+^]_hTM_; the effect is suppressed by HC-06 (n = 43 cells). Averaged trace from a representative experiment. Image acquisition suspended during de-focus (breaks in trace). *Inset:* typical stretch response, shown at 3 sec & 18sec. Scale bar = 10 μm. (**b**) Stretch- [Ca^2+^]_i_ response for cells plated on collagen and Pronectin (fibronectin-like) substrates (N = 5 experiments; n = ≥20 cells/experiment). (**c**) Stretch (12)-evoked [Ca^2+^]_i_ are inhibited by HC-06 (1 μM) and pBPB (100 μM) (N = ≥3 experiments; n = ≥14 cells/experiment). (**d**–**f**) hTM. Stretch promotes TRPV4-dependent formation of actin stress fibers and focal adhesion complexes. Cells labeled for phalloidin-488 and vinculin-ir following stretch (6%, 2 Hz; 1 hr). Stretch-evoked SF upregulation (N = 12 experiments) is antagonized by HC-06 (N = 6) or BAPTA-AM (100 μM; N = 4). Scale bars = 10 or 50 μm, respectively. (**g**) Cyclic stretch (6%; 1 hr) increases FAK phosphorylation, but not overall FAK protein expression (N = 3 experiments). (**h,i**) GSK101 (5 nM, 1 hr) promotes formation of SFs and FAs. This effect is antagonized by HC-06 (N = 3). Scale bar = 50 μm. (**j,k**) Simultaneous imaging of [Ca^2+^]_i_ and actin remodeling. GSK (5 nM) elevates [Ca^2+^]_i_ and stimulates upregulation of mApple-actin –labeled SFs. Numbers denote the time-corresponding images. Scale bars = 10 μm.

Electrophysiological and optical imaging approaches were used to examine TRPV4 function in hTM cells. During voltage ramps (±100 mV over 1 sec), the selective agonists GSK1016790A (GSK101; ~EC_50_ for hTM, ~25 nM; Fig. 3a–c) and 4αPDD (1 μM; Fig. 3d,e) evoked inward and outward transmembrane currents, with outward rectification at positive potentials (e.g., Fig. 3b; n = 9 cells) typical of TRPV4-mediated currents^17^,^19^,^25^. The selective TRPV4 antagonist HC-067047 (HC-06; 1 μM) abolished inward and outward components of the GSK101-evoked current (Fig. 3a,c). HC-06 alone had no effect on baseline currents (Fig. 3c), suggesting that TRPV4 is not constitutively active in hTM cells *in vitro*.

Analysis of fluorescence ratios from hTM cells loaded with the Ca^2+^ indicator dye Fura-2-AM showed that the TRPV4 agonist reversibly elevates [Ca^2+^]_TM_ (Fig. 3f–n). At 25 nM, GSK101 raised [Ca^2+^]_hTM_ to 1.26 ± 0.078 ΔR/R (Fig. 3j), a15.07-fold increase over spontaneous baseline Ca^2+^ fluctuations (base/baseline) (n = 33 cells; P < 0.0001). High-speed imaging revealed the response to the agonist within the first 25 msec (trace 2 in panels 3g & h). Similar TRPV4 responses were observed in pTM cells isolated from a human donor (Fig. 3k,l). Baseline [Ca^2+^]_pTM_ was 28.78 ± 2.09 nM (n = 254 cells) and GSK101 elevated [Ca^2+^]_pTM_ levels to 480.4 ± 54.66 nM (n = 182 cells). Demonstrating specificity, TRPV4 agonist-evoked elevations in [Ca^2+^]_hTM_ (Fig. 3i,j) and [Ca^2+^]_pTM_ (Fig. 3k,l) were abolished by HC-06 (P < 0.0001). HC-06 did not decrease basal [Ca^2+^]_i_ levels, indicating that TRPV4 is not tonically active under these recording conditions.

Agonist-evoked calcium signals typically spread from the plasma membrane to the cell nucleus and remained elevated in the presence of the agonist. To determine the potential contribution of Ca^2+^-induced Ca^2+^ release (CICR), we depleted endoplasmic reticulum Ca^2+^ with cyclopiazonic acid (CPA; 5 μM), an inhibitor of sarcoplasmic-endoplasmic reticulum ATPases (SERCAs). CPA alone elevated [Ca^2+^]_TM_ as Ca^2+^ was released/leaked from the stores but not pumped back in (n = 109 cells; P < 0.01). After store depletion by CPA and cytosolic Ca^2+^ clearance by plasma membrane pumps/exchangers, the amplitude of GSK101 signals was reduced by 39.3 ± 7.99% (P < 0.05) (Fig. 3m). Thus, as observed in other cell types^22^,^26^,^27^, Ca^2+^ release from internal stores amplifies TM plasmalemmal signals mediated by TRPV4.

Endogenous TRPV4 activation was proposed to involve phospholipase A2 (PLA2)- dependent generation of arachidonic acid (AA) and its eicosanoid metabolites^17^. Not all TRPV4-expressing cells utilize this ‘canonical’ mechanism for TRPV4 activation^19^,^28^; yet, the PLA2 pathway is strongly expressed in the TM^29^ and may play a role in glaucoma^30^. To test whether PLA2 signaling plays a role in TRPV4 function, we exposed cells to AA and blockers of cytochrome P450 (CYP450), the downstream enzyme that generates final eicosanoid activators of TRPV4^17^,^22^. 50 μM AA (n = 159 cells) elevated [Ca^2+^]_hTM_ to a extent comparable to 25 nM GSK101 (n = 244 cells; Fig. 3n). AA-evoked calcium responses were blocked by HC-06 (1 μM; P < 0.001; n = 94 cells) and by the pan-CYP450 inhibitor clotrimazole (CTZ, 10 μM; n = 134 cells). Thus, TM TRPV4 channels are potently stimulated by the canonical PLA2 pathway.

TRPV4-mediated signals were proposed to enter TM cytosol primarily through primary cilia^31^. To test this possibility, we exposed hTM cells to GSK101 following 24 hour incubation with the deciliating agent chloral hydrate (4 mM)^32^,^33^,^34^. The compound dramatically reduced the size and prevalence of primary cilia without causing toxicity (Fig. 2c–e), yet GSK101 responses remained robust under these conditions (Fig. 2f–g). These findings suggest that plasma membrane TRPV4 channels (Figs 1c and 2a–c) are sufficient to mediate agonist-evoked [Ca^2+^]_i_ signals TM cells whereas ciliary channels might play minor, auxiliary functions in Ca^2+^ homeostasis^35^.

### Mechanical stretch triggers TRPV4-dependent cytoskeletal remodeling in hTM cells

To test if TRPV4 contributes to mechanotransduction, hTM cells were seeded onto elastic silicone membranes of a StageFlexer device. Periodic, calibrated displacements/relaxations of the collagen I/IV–coated substrate (2 Hz; 2–14% elongation) evoked uniform and reversible elevations in cytosolic [Ca^2+^]_i_ (Fig. 4a). The response amplitude increased with stretch intensity and was comparable between collagen- and Pronectin (fibronectin-like)-coated substrates (Fig. 4b). Most notably, stretch-induced increases in [Ca^2+^]_i_ (n = 124 cells) were suppressed by HC-06 (n = 62; P < 0.001) and the PLA2 inhibitor pBPB (n = 43; P < 0.0085) (Fig. 4c). This indicates that the TRPV4 signaling pathway is required for stretch-induced [Ca^2+^]_hTM_ elevations.

Given that IOP-dependent increases in TM stiffness and contractility involve actin polymerization^9^,^10^, which is regulated by stretch and calcium ions^12^,^14^, we hypothesized that stretch-induced cytoskeletal remodeling might involve TRPV4. Consistent with this, substrate stretch potently stimulated the formation and thickening of phalloidin-Alexa488-tagged F-actin stress fibers (Fig. 4d–f). Stretch also increased phosphorylation of focal adhesion kinases (FAKs; Fig. 4g) and induced reorganization of vinculin, a key FA regulator protein, at the focal ends of stress fibers (Fig. 4d,e). This suggests that mechanical stress both stiffens TM cells and facilitates Ca^2+^-dependent cytoskeletal linkage to the ECM (e.g. ref. 36). Accordingly, pre-incubation of cells with the fast Ca^2+^ chelator BAPTA-AM (100 μM) suppressed the effect of stretch on actin remodeling (P < 0.05) (Fig. 4e,f). The TRPV4 antagonist HC-06 inhibited stretch-evoked [Ca^2+^]_i_ increases (P < 0.005; Fig. 4a,c) and stress fiber upregulation (P < 0.01; N = 7 experiments; Fig. 4e,f), reinforcing the conclusion that TRPV4 activation is required for enhanced cytoskeletal stiffness and reinforcement of contacts between actin and ECM.

We tested whether TRPV4 activation alone is sufficient for mimicking the effect of stretch on cytoskeletal remodeling by exposing the cells to GSK101 (5 nM; 1 hr). As illustrated in Fig. 4h,i, exposure to the TRPV4 agonist triggered stress fiber and FA remodeling that mirrored cytoskeletal changes induced by mechanical strain and was likewise antagonized by HC-06 (P < 0.05; N = 3 experiments; 3–5 slides/experiment). Simultaneous recording of [Ca^2+^]_i_ and mApple-actin dynamics confirmed that TRPV4-induced stress fiber formation takes place during the Ca^2+^ elevation (Fig. 4j,k and Supplementary Video S1).

### TRPV4 inhibition enhances outflow facility and lowers perfusate pressure in 3D pTM cell cultures

**Figure 5:**
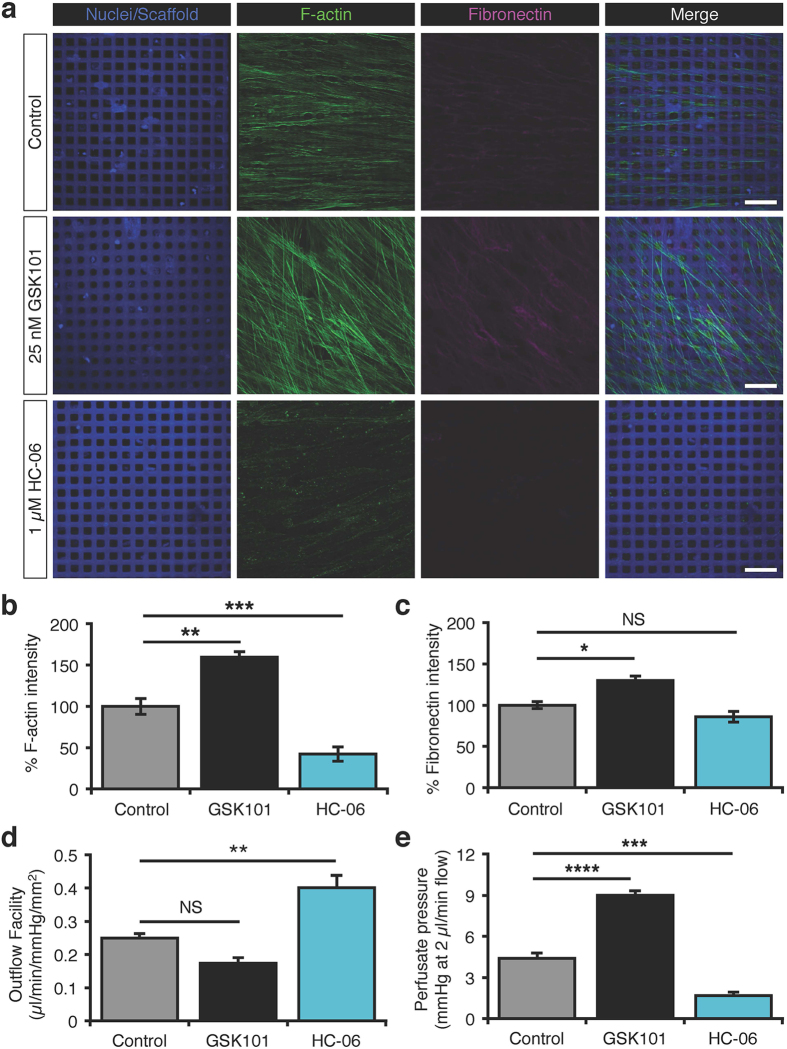
TRPV4 regulates the outflow facility in 3D pTM nanoscaffold models of conventional outflow together with remodeling of the cytoskeleton and extracellular matrix. pTM-seeded SU-8 scaffolds. (**a**) Representative IHC images of F-actin and fibronectin following 24 hrs of perfusion. Scale bars = 30 μm. (**b**) Quantification of F-actin signals from (**a**). (**c**) Quantification of fibronectin signals from (**a**). (**d**) HC-06 (1 μM) increases the outflow the facility in 3D pTM cultures, whereas GSK101 (25 nM) non-significantly attenuates it (N = 5 experiments from 3 donors). (**e**) Relative to the vehicle control, HC-06 lowers perfusate pressure, whereas GSK101 has the opposite effect (N = 5 experiments from 3 donors).

Convenience and accessibility encourage studies of TM cell behavior on 2D surfaces; however, *in vivo* these cells experience 3D microenvironments that alter many aspects of cell behavior, including mechanical constraints. To investigate the potential role of TRPV4 in the regulation of outflow, we used bioengineered 3D TM constructed from pTM cells seeded onto micropatterned, porous SU-8 scaffolds^37^,^38^. pTM cultures from three human donors were placed in a perfusion apparatus with an integrated sensor continuously detecting fluid flux and hydrostatic pressure and perfused to mimic outflow for 6 hours per flow rate (2, 4, 8 and 16 μl/min). After perfusion, cultures were fixed and immunostained to evaluate the cytoskeleton and extracellular matrix. Treatment of pTM-populated scaffolds with GSK101 (25 nM) increased the formation of F-actin stress fibers (P < 0.01) and expression of fibronectin (P < 0.05; Fig. 5a–c), a major component of extracellular matrix deposition in glaucomatous TM that may contribute to outflow obstruction^39^. Conversely, HC-06 (1 μM) reduced levels of stress fibers (P < 0.001) and non-significantly attenuated levels of fibronectin (Fig. 5a–c). Consistent with this, treatment of 3D pTM cultures with GSK101 non-significantly diminished outflow facility (Fig. 5d; P > 0.05; N = 5 experiments), whereas exposure to HC-06 resulted in a dramatic enhancement of the outflow facility (Fig. 5d; P < 0.01; N = 5 experiments). Consequently, GSK101 increased hydrostatic pressure within media perfused through the culture and scaffold at flow rate of 2 μl/minute (Fig. 5e, P < 0.0001; N = 5 experiments), which is comparable to the rate of aqueous outflow in adult human eyes^40^, whereas the TRPV4 antagonist significantly reduced perfusate pressure (Fig. 5e P < 0.001; N = 5 experiments). By showing that TRPV4 activity regulates perfusion pressure-dependent remodeling of the TM, outflow facility and perfusate pressure, these data suggest that inhibition of TRPV4 activity in the TM might promote drainage by the conventional outflow pathway.

### TRPV4 channel activation contributes to pathological increases in IOP

**Figure 6:**
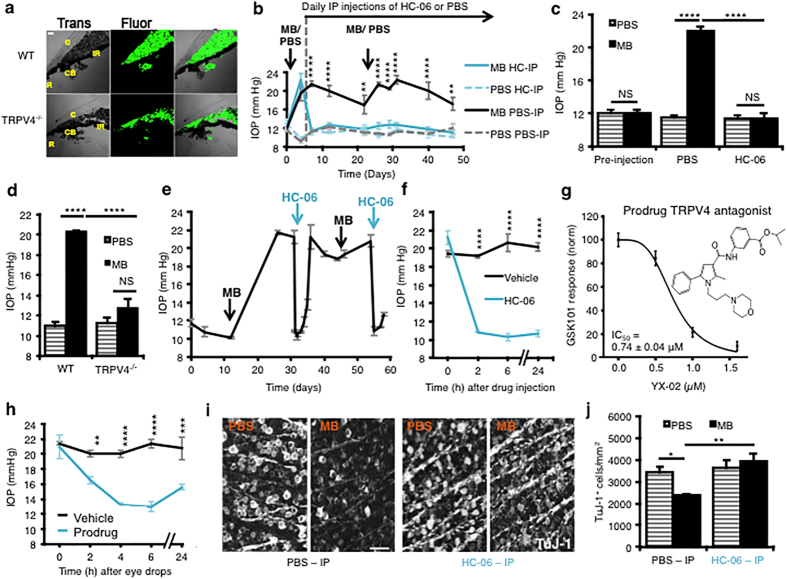
TRPV4 inhibition lowers IOP elevation and protects RGCs in a mouse glaucoma model. (**a**) Injected microbeads clog the anterior chamber in WT and *Trpv4*^−/−^ eyes 7 days post-injection; bright-field transmitted image (*left panel*) and fluorescent image (*middle panel*). Abbreviations: C, cornea; R, retina, CB, ciliary body, IR, iris. Scale bar = 50 μm. (**b**) Intracameral injection of MBs (black trace) but not vehicle (PBS; dashed grey trace) elevates IOP. Subsequent daily IP delivery of HC-06 (solid blue trace) lowered IOP to baseline, whereas vehicle injection had no effect (dashed light blue trace). MBs and PBS were re-injected into the anterior chamber to maintain IOP elevation (second arrow). (**c**) Cumulative data for the effects of MB/PBS injection in the presence/absence of the TRPV4 antagonist 7 weeks post-injection (N = 4 experiments; N = 10–15 animals per experimental group). (**d**) MB injection into WT, but not *Trpv4*^−/−^, eyes (N = 4 mice) elevates IOP above levels in PBS injected eyes. (**e**) Intraocular injection of HC-06 (100 μM) resulted in long-lasting lowering of IOP in MB-injected eyes. (**f**) Intraocular injections at a shorter time scale. (**g**) Chemical structure of the putative prodrug analog YX-02: isopropyl 3-(2-methyl-1-(3-morpholinopropyl)-5-phenyl-1H-pyrrole-3-carboxamido)benzoate (IUPAC). YX-02 dose-dependently suppresses GSK101 (25 nM)-induced Ca^2+^ signals in hTM cells. (**h**) A single topical application of the prodrug was sufficient to decrease IOP for ~24 hours in MB-injected eyes (n = 4 animals) whereas the vehicle (PBS) had no effect. (**i,j**) Retinal wholemounts isolated after 2 months of IOP elevation. The density of Tuj-1^+^ cells in the RGCL was significantly reduced, whereas daily IP HC-06 injection protected the cells from elevated IOP (N = 3 experiments; n = 10–15 animals per experimental group). Scale bar = 50 μm.

To understand the pathophysiological relevance of our *in vitro* observations in eye disease, we investigated the effects of pharmacological and genetic targeting of TRPV4 channels in a widely used mouse model of acute glaucoma^41^. Injecting the anterior eye with polystyrene microbeads (MBs) to obstruct fluid drainage (Fig. 6a,b) elicited a modest IOP increase in mouse eyes (to 20.04 ± 1.24 mm Hg), whereas intracameral injections of the vehicle (PBS) in the contralateral eye had no effect on IOP (9.8 ± 0.26 mm Hg) (Fig. 6b,c). Following IOP elevation, animals from MB- and PBS-treated cohorts were randomly assigned to control or treatment groups for daily intraperitoneal (IP) injections of PBS or HC-06 (10 mg/kg)^25^. Systemic injection of the TRPV4 antagonist lowered IOP to baseline levels (P < 0.0001; 2-way repeated measures ANOVA followed by Holm-Šídák tests in Fig. 6c; 2-way ANOVA followed by Tukey’s test in Fig. 6c). Daily HC-06 treatment maintained low IOP levels in MB-treated eyes, which remained indistinguishable from vehicle-treated eyes. The IOP in age-matched, uninjected, WT eyes (12.84 ± 0.89 mm Hg; n = 25)was similar to the IOP in *Trpv4*^−/−^ eyes (12.40 ± 0.88 mm Hg; n = 50; p > 0.05), indicating that TRPV4 activity during physiological conditions is not a major regulator of IOP. MB microinjection failed to elevate IOP in 4/4 *Trpv4*^−/−^ animals above levels in PBS-injected eyes (Fig. 6d) showing that *Trpv4*^−/−^eyes are resistant to pressure elevation induced by partial angle blockage. These data indicate that overactivation of the TRPV4 channel is necessary to sustain ocular hypertension.

We further tested the efficacy of HC-06 by injecting it (in 1.85% DMSO and PBS, 2 μl) directly into the anterior chamber of eyes with elevated IOP from MB injections (Fig. 6e,f). Single intraocular HC-06 injections induced a dramatic lowering of IOP that lasted for days (Fig. 6e), whereas injection of the vehicle did not lower IOP (Fig. 6f; P < 0.0001; Holm-Šídák tests). To test whether IOP can be controlled through topical delivery, we designed and synthesized a putative prodrug analog (YX-02) of the antagonist HC-06 (Fig. 6g). Confirming efficacy as a TRPV4 antagonist, YX-02 inhibited 25 nM GSK101-induced [Ca^2+^]_hTM_ elevations with an IC50 of 0.74 ± 0.04 μM (Fig. 6 g). A single eye drop of YX-02 decreased IOP to baseline levels for up to 24 hrs (Fig. 6 h; P < 0.01 to P < 0.0001; Holm-Šídák test). HC-06 or YX-02 administration evoked no obvious ocular inflammatory or toxicity response.

8 weeks after initiation of the IOP elevation protocol, wholemount counts of TuJ-1^+^ cells in the retinal ganglion cell layer (RGCL)/mm^2^ were 3425.1 ± 251.3 in vehicle-treated eyes and 2370.1 ± 72.8 in MB-treated eyes, significantly lower (p < 0.05). MB-injected eyes of mice treated daily with systemic HC-06 were protected from the deleterious effects of IOP-elevation across all retinal quadrants (p < 0.01; Fig. 6i,j).

## Discussion

Mechanical stimuli impact every cell in the vertebrate eye and sensitivity to force is essential for visual function. However, the mechanisms by which ocular cells sense and transduce mechanical forces are incompletely understood, affecting our insight into the physiology of IOP homeostasis and treatment of glaucoma. This question is particularly compelling with respect to the conventional outflow pathway because — possibly as a result of structural changes caused by mechanical stress — TM resistance to fluid flow increases dramatically during pathological IOP elevations^2^,^3^,^4^,^5^. In this study, we present evidence that TRPV4 is critically involved in the transduction of mechanical stress in TM cells and link overactivation of this force-sensitive channel to maintaining elevated IOP in a mouse model of glaucoma.

Emerging evidence supports a role for Ca^2+^-initiated signals in increased resistance of the conventional pathway to fluid outflow^9^,^13^; however, neither the identity of the Ca^2+^ channel involved nor the mechanism by which Ca^2+^ signals promote these effects have been fully elucidated. We demonstrate the presence of *Trpv4/TRPV4* transcripts, protein, currents and/or Ca^2+^ signals in primary and immortalized cells from human TM preparations and in *ex vivo* mouse and human tissue. Our evidence suggests that TRPV4 mediates force-induced elevations in [Ca^2+^]_TM_ and reorganization of mechanically stressed TM. Consistent with this, force application to TM cells evoked sustained, stretch-dependent Ca^2+^ elevations that were mimicked by GSK101 and suppressed by HC-06.

Mechanical stretch of TM cells triggered the formation, thickening and cross-linking of F-actin stress fibers, phosphorylation of FAK, and vinculin recruitment to focal adhesions. While this response might involve additional force sensors^42^, the sensitivity of stretch-induced cytoskeletal reorganization and/or [Ca^2+^]_i_ signals to BAPTA-AM, PLA2 blockers and HC-06 clearly shows that these processes involve TRPV4-dependent influx of Ca^2+^. Our data thus identify a novel Ca^2+^ source which drives actomyosin remodeling associated with increased TM stiffness/contractility in response to mechanical stress^9^,^14^,^43^, whereas Ca^2+^-dependent proteins (such as K^+^ and Cl^−^ channels and/or myocilin) might exert additional modulating influence. Similar effects of TRPV4 activation on actin and contractility have been reported in other cell types that regularly experience hydrostatic pressure and cyclic strain^44^,^45^,^46^. We hypothesize that TRPV4-mediated Ca^2+^ signals and/or TRPV4 coupling to actin^14^,^47^, integrin:collagen, β-catenin:E-cadherin^44^ and/or TRPV4:RhoA/Rho kinase mechanisms^48^,^49^ regulate the permeability of juxtacanalicular TM^46^,^47^,^48^,^49^. By analogy with similar roles for the channel in epithelial and endothelial tissues^24^,^44^,^45^,^46^,^48^, we hypothesize that inhibition of TRPV4-induced [Ca^2+^]_i_ elevations suppresses the transfer of force from integrins to focal adhesions and actin, and disrupts stretch-induced polymerization of actin. Accordingly, we discovered that TRPV4 inhibition lowers the density of actin stress fibers (Figs 4 and 5), weakens β-catenin-containing cell-cell contacts and increases paracellular permeability^44^. These results suggest that the TM mechanosensor is integrated into dynamic modulation of stress fibers, which have been proposed to underlie increased TM rigidity, contractility and resistance to aqueous outflow^9^,^10^,^14^.

**Figure 7:**
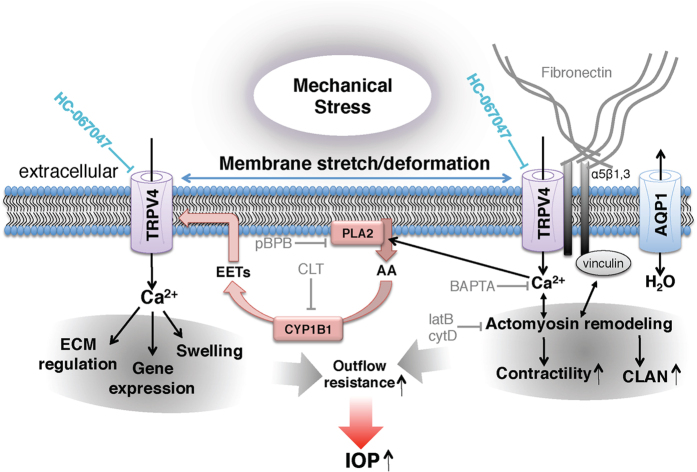
Model of trabecular meshwork signaling in response to mechanical stress. Mechanical stress (e.g., pressure, swelling, and tissue distension) stretches the plasma membrane and activates TRPV4 and a Ca^2+^- and stretch-sensitive phospholipase A2 (PLA2). The product, arachidonic acid (AA), is a substrate for cytochrome P450, which drives synthesis of eicosanoid metabolites (EETs), the final activators of TRPV4. Stretch might activate PLA2 simultaneously with TRPV4; alternatively, stretch-induced TRPV4 activation could stimulate Ca^2+^-dependent PLA2s which amplify the initial TRPV4 signal (horizontal blue arrows). TRPV4 activation may be additionally augmented by TRPV1 channels and/or cell swelling, mediated through aquaporin 1 (AQP1) channels^67^,^68^,^69^. Stretch regulates the outflow resistance through Ca^2+^ -dependent actin remodeling, focal adhesion stabilization, actomyosin contractility, fibronectin production and TM gene expression. Acting in concert, these signaling components account for many known aspects of the TM response to mechanical and inflammatory challenges, including the response to the response to chronic IOP elevations.

The hypothetical mechanism depicted in Fig. 7 shows TRPV4 as a central mediator of stretch- and Ca^2+^-dependent gene/cytoskeletal remodeling in TM cells. We propose that TRPV4 stimulation drives the upregulation of crucial ECM components (collagen and fibronectin), linking diverse types of mechanical stress (IOP, swelling, stretch) to the internal cellular strain-sensing machinery (based on integrins and focal adhesions), and release of AA^50^. This model is supported by our discovery that TRPV4 activation upregulates SF formation and secretion of fibronectin while TRPV4 inhibition prevents strain-induced [Ca^2+^]_i_ elevations and TM remodeling, and reduces perfusate pressure in 3D scaffolds bioengineered with human TM. The dramatic IOP lowering induced by topical or systemic application of TRPV4 blockers in our animal glaucoma model was achieved by breaking the link between TRPV4 activation and activation of downstream calcium-dependent signaling pathways. Given that TRPV4 overactivation contributes to two other key hallmarks of glaucoma pathology —RGC injury and reactive gliosis, both of which are reduced when retinas are subjected to systemic TRPV4 inhibition^21^,^22^,^23^, we propose that therapy using TRPV4 antagonists might achieve the hitherto unachievable trifecta in glaucoma treatment by controlling IOP protecting RGCs from mechanical stress and suppressing inflammatory activation of retinal glia.

It is important to compare our findings with evidence that baseline IOP is slightly elevated in *Trpv4*^−/−^ mouse eyes and that activation of TRPV4, confined to the ciliary membrane of pTM cells, lowers basal IOP in mice and the *Wpk* rat^31^. Our data in contrast suggest that resting IOP is comparable in WT and *Trpv4*^−/−^ mouse eyes and is unaffected by intraocular injections of GSK101^24^, whereas pharmacological inhibition and genetic disruption of TRPV4 were remarkably effective in lowering IOP during partial outflow obstruction. The main implication of these results is that TRPV4 inhibition enhances the outflow facility when outflow resistance is increased. We hypothesize that this facilitation effect is due to decreased cytoskeletal/FA/ECM remodeling that is downstream from pressure-induced TRPV4 activation. Our data also suggest that the conventional outflow pathway retains enough hydraulic conductivity (possibly in collaboration with uveoscleral outflow) to maintain fluid transport in the presence of microbeads and HC-06. Although we cannot exclude the possibility that the dramatic IOP lowering observed *in vivo* included HC-06-dependent facilitation of uveoscleral drainage, hydraulic conductivity of the Schlemm’s canal or attenuated aqueous production, the dramatic potentiation of the outflow facility observed in biomimetic pTM-populated scaffolds demonstrates that suppression of the putative TM mechanosensor is in itself sufficient to stimulate outflow facility.

Importantly, our data demonstrate that both mechanical stress and TRPV4 activation strengthen the cells’ intrinsic tensile apparatus composed of actin stress fibers, focal adhesions and extracellular matrix. Because pressure-induced increases in TM resistance to aqueous outflow require cell stiffening and actin upregulation^14^,^43^,^51^,^52^,^53^,^54^, it is possible that targeting TRPV4 counters the effect of mechanical stress by ‘protecting’ the downstream transduction mechanism. Cyclic stretch increased secretion of fibronectin in pTM-populated nanoscaffolds, in pulmonary cells (which continually experience) tensile stretch^55^ and in glaucomatous TM^7^. We conjecture that *in vivo* IOP elevations impose tensile strain onto ECM, inducing influx of Ca^2+^ through stretch-sensitive TRPV4 channels. The resulting increase in [Ca^2+^]_i_ triggers changes in gene expression, reorganization of the TM cytoskeleton and secretion of ECM that ultimately impede fluid passage through the delicate meshwork formed by TM processes (Fig. 7). Because TRPV4-incompetent cells lack the mechanism that couples force to the cytoskeletal/matrix they cannot sustain IOP elevations in response to microbead injection. Consistent with our model, elevated pressure disproportionally increases cytosolic Ca^2+^ levels^11^,^13^ and induces TM stiffening in POAG TM cells^9^,^10^,^43^,^54^. Stretch tended to be a more effective facilitator of stress fiber formation than GSK101 even when both stimuli were applied at a ~half-maximal dose (6% stretch vs. 25 nM) for [Ca^2+^]_i_ elevations. Because HC-06 and BAPTA-AM were equally effective in antagonizing the effects of stretch/TRPV4 agonists on actin remodeling, we propose that the former activates auxiliary (yet TRPV4-dependent) downstream signaling mechanisms (possibly involving the Rho signaling pathway), which are less effectively stimulated by chemesthesis alone. This is consistent with the observation that TRPV4-activating stimuli promote channel opening through different amino acid residues of the protein^18^,^56^.

While our report suggests a critical role for TM TRPV4 in sustaining IOP elevations, other ocular sites may also be impacted by TRPV4 antagonists or channel deletion. For example, HC-06 could affect the function of nonpigmented epithelial cells of the ciliary body^24^,^57^, corneal endothelial cells^58^, retinal ganglion cells^23^ as well as inflammatory signals mediated through glial cells^22^,^28^; however, the absence of antagonist/agonist/knockout effects on baseline IOP argues against a major role for TRPV4 in steady-state fluid secretion or drainage. An important question to be addressed in future studies is whether and how these TRPV4-expressing ocular tissues might respond to physiological fluctuations in IOP induced by saccades, changes in posture, etc. TRPV4 channels could also play a role in the modulation of aqueous humor transfer through transcellular or paracellular routes in the inner wall of Schlemm’s canal, possibly by regulating tight/*adherens* junctions of cells which functionally resemble TRPV4-containing vascular endothelia^8^,^46^,^59^. However, our data show that primary cilia are not required for TRPV4 signaling in immortalized or primary human TM cells, and are thus consistent with evidence against a role for primary cilia in cytosolic Ca^2+^ homeostasis^35^ and TRPV4 signaling^33^,^60^. Antibody staining, optical imaging and use of deciliating agents showed that the large majority of TRPV4 channels are localized to the TM cell plasma membrane and may be functionally coupled to CICR and, possibly, store-operated calcium signaling^61^.

Our observations that PLA2 antagonists inhibit stretch-induced Ca^2+^ signals and that TRPV4 blockers suppress AA-induced [Ca^2+^]_TM_ increases suggest that the channel is activated through the canonical pathway. It remains to be determined whether PLA2 functions as the primary force sensor or a TRPV4-mediated rise in [Ca^2+^]_TM_ might stimulate translocation, membrane binding and phosphorylation of cPLA2, which cleaves phospholipids at the *sn*-2 position to generate AA, the precursor of eicosanoid activators of TRPV4 (Fig. 7). The involvement of proinflammatory eicosanoids could, over time, exacerbate the pathological response to mechanical stress. It is thus of interest that PLA2 is conspicuously upregulated in POAG TM^29^ and that mutations in the TM-specific B1 isoform of cytochrome P450 were associated with early-onset glaucoma^30^. Further consistent with our model, PLA2 blockers mirror the effect of TRPV4 antagonists on IOP by inhibiting actin stress fiber formation and linkage to the ECM^62^.

In summary, our data bring together previously unrelated aspects of force sensing by TM cells and place them within a novel mechanistic framework that may provide insight into the molecular linkage between mechanotransduction, Ca^2+^ homeostasis and reorganization of the conventional outflow pathway. Given that IOP reduction represents a highly effective treatment of both hypertensive and normal-tension versions of the disease^1^, the novel prodrug analog described here may provide safe and effective glaucoma therapy by targeting the pressure sensor, a major currently unmet need in the clinical treatment of glaucoma^2^. The potential for protecting RGCs from mechanical stress by TRPV4 inhibition^21^,^63^ offers additional impetus to explore possible solutions for combined IOP-lowering and neuroprotective treatments to prevent vision loss in glaucoma.

## Materials and Methods

### Study approval

Experiments followed recommendations of the NIH Guide for Care and Use of Laboratory Animals and the ARVO Statement for the Use of Animals in Ophthalmic and Vision Research. All animal studies were approved by the Institutional Animal Care and Use Committee (IACUC) of the University of Utah.

### Animals

C57BL/6J mice were from JAX (Bar Harbor, ME) and *Trpv4*^−/−^ mice were obtained from Dr. Wolfgang Liedtke (Duke University). Mice were maintained in a pathogen-free facility with a 12-hour light/dark cycle and *ad libitum* access to food and water. Temperature was set at ~22–23 °C. Up to 5 adult mice of the same gender were housed in a single cage. Animals were regularly monitored during and following surgical procedures and were promptly euthanized in rare instances of declining health. Male and female mice were included in the study. No sex differences in the data were noted, so their data were pooled. Mice were 3 to 6 months in age. Sample sizes were based on pilot experiments. An independent co-author randomized group assignment and coded conditions to blind experimenters to conditions involving animals.

### TM cell culture and isolation

Primary hTM cells, isolated from the juxtacanalicular and corneoscleral regions of the human eye (ScienCell Research Laboratories; Carlsbad, CA) were grown in Trabecular Meshwork Cell Medium (ScienCell, Catalog#6591) at 37 °C and 5% CO_2_. Confluent cells showed the flattened phenotype that is typical of cultured hTM. To test whether hTM cells exhibit molecular and physiological (steroid sensitivity) characteristics typical of primary TM cultures, we measured expression of αB-crystallin, TIMP3, aquaporin 1 and smooth muscle actin genes and dexamethasone (DEX)-induced expression of myocilin (Supplementary Fig. S1). Key physiological features (e.g., responses to TRPV4 agonists) were replicated in primary TM (pTM) cells isolated from two donors (35 and 40 years old) and cultured following established protocols. Human corneal rims used to culture primary TM cells were obtained by Dr. Balamurali Ambati (Moran Eye Institute, University of Utah). Human tissues were used in concordance with the tenets of the WMA Declaration of Helsinki and the Department of Health and Human Services Belmont Report. Salts and reagents were purchased from Sigma or ThermoFisher unless specified otherwise.

### mRNA and protein analysis

Total RNA was extracted from hTM cells using E.Z.N.A total RNA kit (Omega). RNA was reverse transcribed using the XLT cDNA super mix kit (Quanta). Amplified *Trpv4* mRNA was referenced to *α-tubulin* signals for each sample. For immunoblots, hTM cells were dissociated from culture flasks and centrifuged at 2000 rpm for 3 minutes. The cell pellet was washed with PBS and homogenized in 20 mM Tris buffer (pH 7.4) containing: 0.1 M NaCl, 0.2 mM EDTA, 0.2 mM PMSF, 50 mM NaF and the proteinase inhibitor cocktail (Santa Cruz). Protein was separated by 10% SDS-PAGE gels and transferred to polyvinylidene difluoride membranes (Bio-Rad). Membranes were blocked with 5% skim milk in PBS containing 0.1% tween 20 and incubated at 4 °C overnight with primary antibodies against myocilin (rabbit polyclonal, 1:500; Sigma 276–290), FAK (rabbit polyclonal 1:1000; Cell Signaling Technology 3285S) or pFAK-(Tyr397) (rabbit polyclonal 1:1000; Cell Signaling Technology 3283S). Secondary antibodies were conjugated to horseradish peroxidase and visualized using on X-ray film (Thermo Scientific) by using ECL solution (Pierce) and a developer machine (AFP Imaging Corp).

### Cryosection immunohistochemistry

Mice were deeply anesthetized with isoflurane and transcardially perfused with PBS, followed by 4% PFA in PBS. Eyes were postfixed for 2 hrs at 4 °C and rinsed with PBS. Fresh TM tissue explants were fixed in 4% PFA and PBS for 1 hr at room temperature (RT). Tissue was cryoprotected in 15% (1 hr RT) and 30% sucrose (4 °C overnight) and embedded in OCT (Tissue-Tek). 12–16 μm slices were cut with a cryostat and mounted on Superfrost plus slides (Fisher Scientific). Slides were dried and frozen at −80 °C until use. Slices were rinsed in PBS, permeabilized and blocked (1% BSA, 0.3% Triton X-100/PBS and 0.1% NaN_3_) for 30–60 minutes at RT. Primary antibodies (rabbit anti-TRPV4, 1:1000, Lifespan C94498; mouse anti-β-catenin 1:1000, a kind gift of M.E. Hartnett, University of Utah; mouse anti-α-tubulin, 1:1000, Sigma T6793; mouse anti-vinculin, 1:500, Sigma HVIN-1; Wheat Germ Agglutinin Alexa-Fluor 594 conjugate, 1:200, ThermoFisher W11262) and secondary antibodies (goat anti-rabbit Alexa Fluor 488, 1:1000, ThermoFisher A-10680; goat anti-mouse Alexa Fluor 594, 1:1000, ThermoFisher A-11005) were diluted in blocking solution and applied for 12 hrs (4 °C) and  2 hrs (RT), respectively. Anti-TRPV4 antibodies from Alomone Laboratory and Santa Cruz Biotechnology produced non-specific signals. Cell nuclei were counterstained with DAPI in mounting solution (DAPI-fluoromount-G, Southern Biotech) and coverslipped.

To assess changes in cytoskeletal remodeling in hTM by GSK101, cells were plated on type I collagen-coated glass coverslips for 24 hrs and were then treated with DMSO (control group), 5 nM GSK101, 1 μM HC-06 or 1 μM HC-06 and 5 nM GSK101 (HC-06 was added 30 min before GSK101) for 1 hr at 37 °C. To examine cytoskeletal remodeling, cultures were fixed in 4% PFA, washed twice in PBS, permeabilized with 0.1% Triton X-100, washed twice in PBS and exposed to blocking solution. F-actin was labeled with phalloidin-Alexa 488 nm or phalloidin-Alexa 594 nm, either alone or in the presence of another antibody. Cells were washed twice in PBS and mounted with DAPI-Fluoromount-G (Southern Biotech). The distribution of F-actin and vinculin was determined in confocal (Olympus CV1200) stacks using NeoFluor 40x water immersion objectives. Formation of stress fibers was quantified by averaging the overall fluorescence within the field of view of 30 ROIs per experiment. Final data contains averaged data from at least 3 separate experiments. Experimenters were blinded to the condition during imaging and analysis.

### Electrophysiology

Macroscopic whole-cell currents were recorded using a Multiclamp 700B amplifier and digitized by DigiData 1550 (Molecular Devices). Data were sampled at 10 kHz and filtered at 5 kHz. Whole-cell current was elicited by −100 mV to 100 mV ramps from the holding potential of −70 mV. RAMP pulses were of 1 sec duration and applied at 0.2 Hz. The standard extracellular recording solution contained (in mM): 150 NaCl, 3 KCl, 1 MgCl_2_, 1.8 CaCl_2_, 10 HEPES, 5.5 D-glucose. The pipette solution contained (mM): 135 K-gluconate, 10 KCl, 10 HEPES, 1 MgCl_2_, 4 Mg-ATP, 0.6 Na-GTP, 2 mM BAPTA. pH was adjusted to 7.3 with KOH and osmolarity was adjusted to 296–300 mOsm with sucrose. All experiments were performed at RT (22–23 °C).

### Calcium imaging

Cultured hTM/pTM cells were loaded with 5 μM Fura-2-AM for 15–30 minutes and were perfused with isotonic saline (pH 7.4) containing (in mM): 98.5 NaCl, 5 KCl, 3 MgCl_2_, 2 CaCl_2_, 10 HEPES, 10 D-glucose, 93 mannitol. Epifluorescence imaging was performed as described^22^,^23^ using inverted Nikon Ti or upright Nikon E600 FN microscopes with 20x (0.75 N.A. oil) and 40x (1.3 N.A. oil & 0.8 N.A. water) objectives and Nikon Elements software. In a subset of experiments, [Ca^2+^]_pTM_ levels were calibrated using the standard equation (K_d_ at RT = 224 nM). Results represent averages across cells (3–6 slides, each containing 20–40 cells) from at least three separate experiments.

### Live actin imaging

hTM cells were transfected with a mApple-actin DNA construct using Lipofectamin (Invitrogen). After 24 hrs, cells were loaded with Fura-2-AM (5 μM) for 45 minutes. mApple-actin (a kind gift from Dr. Mary Beckerle, University of Utah) and Fura-2 fluorescence were concurrently imaged with a 40x/1.4 NA objective and appropriate excitation and emission filters.

### Cell stretch assay

hTM cells were plated on flexible silicon membranes coated with type I/IV collagen or Pronectin (protein polymer incorporating multiple copies of the RGD attachment epitope from human fibronectin) and grown to 80% confluence. Cells were placed into a FlexJunior chamber controlled by the Flexcell-5000 Tension system (Flexcell, Hillsborough, NC) and stimulated with cyclic biaxial stretch (6%, 2 Hz) for 1 hour at RT. 1 μM HC-06 or the vehicle were added 1 hr prior stretching^65^,^66^. To chelate cytosolic Ca^2+^, cells were loaded with 100 μM BAPTA-AM for 30 minutes prior stretching. Then samples were used to analyze cytoskeletal remodeling as described above. For determination stretch-induced Ca^2+^ influx, cells were loaded with Fura-2-AM for 30–60 minutes and then stimulated with cyclic biaxial stretch (2–14%, 2 Hz). A 5 minute stretch period was chosen to optimize capture of the stretch-evoked fluorescent response by adjusting for the change in focal plane (which disrupted calcium imaging for several seconds as indicated by breaks in response trace in Fig. 4a). Cells were imaged with a Nikon E600FN upright microscope. Excitation light was provided by a xenon lamp within a Lambda DG4 (Sutter Instruments) controlled by Nikon Elements.

### Bioengineered TM: 3D culture of primary human TM cells on scaffolds

Primary TM cells were isolated from donor tissue rings discarded after penetrating keratoplasty. Before use, all pTM cell strains were characterized for expression of αB-crystallin and α-smooth muscle actin. pTM cells were initially plated in 75 cm^2^ cell culture flasks with 10% fetal bovine serum (FBS) (Atlas Biologicals, Fort Collins, CO) in Improved MEM (IMEM) (Corning Cellgro, Manassas, VA) with 0.1 mg/mL gentamicin. Fresh medium was supplied every 48 hrs. Cells were maintained at 37 °C in a humidified atmosphere with 5% carbon dioxide until 80–90% confluence, at which point cells were trypsinized using 0.25% trypsin/0.5 mM EDTA (Gibco, Grand Island, NY) and subcultured. All studies were conducted using cells before the 5th passage. To create 3D pTM constructs, 40,000 pTM cells were seeded on individual microfabricated SU-8 scaffolds placed in a 24-well plate and cultured in 10% FBS-IMEM for 14 days. Medium was changed every 2–3 days. On day 14, 3D pTM constructs were serum-starved for 24 hrs and then treated with GSK101 (25 nM), HC-06 (1 μM) or the vehicle for 24 hrs.

### Perfusion of bioengineered TM

Samples were securely placed in a perfusion chamber^37^,^38^ and media was perfused through the culture/scaffold for 6 hrs per flow rate (2, 4, 8 and 16 μl/min). Samples were perfused in an apical-to-basal direction with perfusion medium consisting of Dulbecco’s modified Eagle’s medium (DMEM) (Cellgro) with 0.1% gentamicin (MP) containing GSK101 (25 nM), HC-06 (1 μM) or the vehicle. The temperature was maintained at 34 °C. Pressure was continuously monitored and recorded. After perfusion, samples were fixed and stained for confocal imaging. The “outflow facility” of our bioengineered 3D pTM model, Δflow/Δpressure, was calculated from the inverse of the slope of the pressure versus flow graph per unit surface area. These experiments were performed with the experimenter blinded to the compound identity.

### Microbead occlusion glaucoma model

Mice were anesthetized with an intraperitoneal (IP) injection of ketamine/xylazine (90 mg/10 mg / kg of body weight) and eye drops were used to numb the eyes (0.5% proparacaine hydrochloride) and dilate the pupils (1% tropicamide ophthalmic solution USP; Bausch & Lomb). IOP was elevated unilaterally as in^64^ by injecting 2 μl of polystyrene microbeads (7.8 μm FluoSpheres; Bangs Laboratories) with a blunt tip, Hamilton syringe (Hamilton Company) into the anterior chamber after making a guide hole using a 30.5 gauge needle, gently depressing the cornea to displace aqueous humor and drying the eye. Microbeads were injected over 60 s and the needle remained for an additional 60 s before injecting a small bubble to seal the cornea and prevent microbead outflow. The contralateral eye was injected with PBS. 0.5% Erythromycin ophthalmic ointment USP (Bausch & Lomb) was applied after the procedure. Injected eyes were visually examined for MBs at multiple time points during experiments. Following injections, intracameral MBs were stably localized to the angle. This procedure and IOP measurements were performed in a hood within the Moran Eye Center vivarium.

### Mouse IOP measurements

A TonoLab rebound tonometer was used to measure IOP of Avertin-treated (Fig. 6b–c) and awake mice (Fig. 6d–f,h)^64^ between 10 AM and noon. For awake mice, 0.5% proparacaine hydrochloride was applied prior to measurements. IOP was determined from the mean of 10 to 20 tonometer readings.

### *In vivo* TRPV4 inhibition and generation of prodrugs

HC-067047 (Sigma Aldrich, St. Louis, MO) was dissolved in 1.85% DMSO and PBS, then it or the vehicle was administered via a 10 mg/kg intraperitoneal injection (IP) once daily for 8 weeks. For topical application, the prodrug was identical to HC-06 except an isopropyl ester group replaced the trifluoromethyl group. To make the prodrug (Fig. 6f), 2-methyl–1-(3- morpholinopropyl)-5-phenyl-1H-pyrrole-3-carboxylic acid (200 mg; 0.610 mmol) in 5 mL dichloromethane (DCM) was added thionyl chloride (220 μL; 3.05 mmol) and dimethylformamide (20 μl). After stirring for 3 hrs at RT, the reaction mixture was evaporated and dried under vacuum pressure. To the residue was added DCM (5 ml), isopropyl 3-aminobenzoate (218 mg; 1.20 mmol) and N,N-diisopropylethylamine (0.42 ml), and the reaction mixture was stirred overnight. Water was added and the mixture was extracted with DCM. The organic phase was washed with water, brine, dried by anhydrous sodium sulfate, concentrated and purified by chromatography to provide the prodrug YX-02 (290 mg; 0.592 mmol, 97% yield). H-NMR (CD3OD/400 MHz): δ 8.32 (s, 1H); 7.89 (d, 1H); 7.71 (d, 1H); 7.42 (m, 6H); 6.65 (s, 1H); 5.21 (m, 1H); 4.07 (m, 2H); 3.54 (m, 4H); 2.64 (s, 3H); 2.15 (m, 6H); 1.64 (m, 2H); 1.38 (d, 6H). MS (ES+, m/z): 490.1 (M++1, 100.0). One drop of a 1.862 μM prodrug solution (1.85% DMSO and PBS) was applied unilaterally to each mouse eye. The vehicle was applied contralaterally as a control.

### Statistical Analysis

GraphPad Prism 6.0 and Origin Pro 8.5 were used for statistics. Means are shown ± SEM. Unless specified, an unpaired t-test was used to compare two means and an ANOVA along with Tukey’s multiple comparisons test was used to compare three or more means^22^,^23^,^69^. P > 0.05 = NS, P < 0.05 = *, P < 0.01 = **, P < 0.001 = *** and P < 0.0001 = ****.

## Additional Information

**How to cite this article**: Ryskamp, D. A. *et al*. TRPV4 regulates calcium homeostasis, cytoskeletal remodeling, conventional outflow and intraocular pressure in the mammalian eye. *Sci. Rep.* **6**, 30583; doi: 10.1038/srep30583 (2016).

## Supplementary Material

Supplementary Information

Supplementary Video S1
