# Supplementary material for: TRPV4 regulates calcium homeostasis, cytoskeletal remodeling, conventional outflow and intraocular pressure in the mammalian eye
Source: Sci Rep. 2016 Aug 11;6:30583. doi: 10.1038/srep30583 (PMC4980693; doi:10.1038/srep30583)
Supplement: Supplementary Information [file srep30583-s1.pdf]

Supplementary Information

Daniel A. Ryskamp, Amber M Frye, Tam T.T. Phuong, Oleg Yarishkin, Andrew O. Jo, Yong Xu, Monika Lakk, Anthony Iuso, Sarah N. Redmon, Balamurali Ambati, Gregory Hageman, Glenn D. Prestwich, Karen Y. Torrejon & David Križaj: **TRPV4 regulates calcium homeostasis, cytoskeletal remodeling, conventional outflow and intraocular pressure in the mammalian eye**

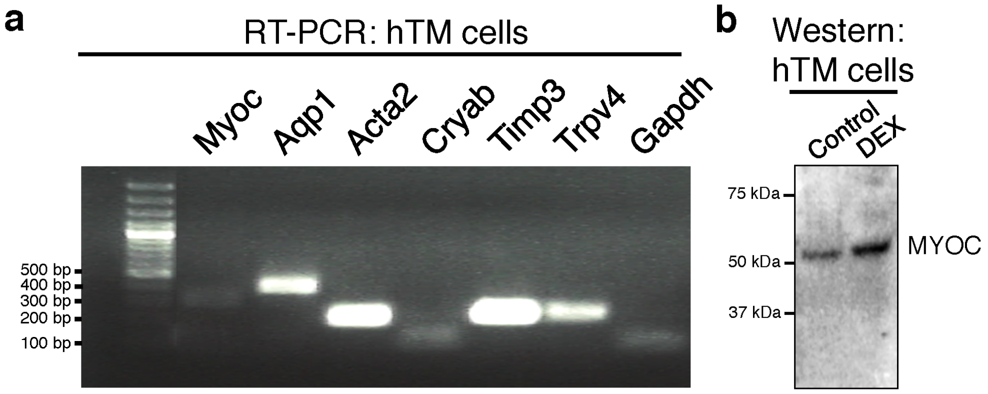

c

Primer	Sequence (5' to 3')	NCBI Reference Sequence	PRC product
**hMyoc** sense	GACACCGGATGTGGAGAACTA	NM_000261.1	299bp
**hMyoc** antisense	GACTCAGCGCCCTGGAAATA		
**hAqp1** sense	TTTGGGTATGAAGCCGTGTC	NM_001185061.1	391bp
**hAqp1** antisense	AGTCAATAGCCAGGAGGTGT		
**hActa2** sense	TTCGCATCAAGGCCCAAGAA	NM_001141945.1	219bp
**hActa2** antisense	GCTTCACAGGATTCCCGTCTTA		
**hCryab** sense	AAACGGCCTGGGTGGATAGA	NM_001885.2	100bp
**hCryab** antisense	CACTGAGCTCAAGACACACTTG		
**hTimp3** sense	CAAGATGTACACGGGGCTGT	NM_000362.4	218bp
**hTimp3** antisense	AGGCGTAGTGTTTGGACTGG		
**hTRPV4** sense	TCCCATTCTTGCTGACCCAC	NM_147204.2	217bp
**hTRPV4** antisense	AGGGCTGTCTGACCTCGATA		
**hGapdh** sense	CTCCTGTTCGACAGTCAGC	NM_002046.5	89bp
**hGapdh** antisense	GACTCCGACCTTCACCTTC		

Supplementary Figure 1. Validation of cultured TM cells. (*A*) Expression of transcripts for TM-specific marker genes in cultured hTM cells. Expression of mRNAs coding for *Myoc*, *Aqp1*, *Acta2*, *Timp3*, *Trpv4* and *Gapdh* was at the expected amplicon sizes. (*B*) Western blot

with an anti-myocilin antibody. Exposure of cell cultures to 100 nM DEX for 2 days upregulated myocilin expression in hTM cells. (*C*) PCR primer information.

Supplementary Video 1. Simultaneous imaging of GSK101-induced mApple:actin remodeling and $[Ca^{2+}]_i$ dynamics in a representative time-lapse of a Fura-2 loaded hTM cells.
